# The protocol for developing health and disease prevention services: An exercise-based prediction model integrating genomic test results

**DOI:** 10.1371/journal.pone.0327947

**Published:** 2025-07-22

**Authors:** Yunsuk Choi, Hyunseok Jee

**Affiliations:** School of Kinesiology, Yeungnam University, Gyeongsan, Gyeongbuk, South Korea; PLOS: Public Library of Science, UNITED STATES OF AMERICA

## Abstract

**Background:**

Cancer is a leading cause of mortality worldwide, with approximately 19.6 million new cases and 10 million deaths reported in 2020. Exercise interventions have demonstrated positive effects on physical and mental health in cancer patients, yet there is limited evidence on the efficacy of tailored, high-intensity exercise programs designed using genomic data. This protocol outlines a study aimed at integrating genomic analysis and personalized exercise interventions to improve health outcomes and reduce cancer-related risk factors. This study aims to evaluate the feasibility and potential impact of a personalized exercise intervention delivered through the EXESALUS mobile application. The program integrates genomic information to tailor exercise regimens for cancer prevention, muscle strength improvement, and quality-of-life enhancement.

**Methods:**

This is a protocol for a 3-month, parallel-group, randomized controlled trial involving 500 participants, including 100 cancer patients undergoing treatment or rehabilitation and 300 non-cancer participants with elevated disease risk. Participants will engage in the EXESALUS program, which includes low-, moderate-, and high-intensity exercise tailored to genomic profiles, supported by exercise counseling and wearable device feedback. Biospecimens (blood, urine, and oral epithelial cells) will be collected at baseline, 6 weeks, and 3 months to assess genomic variations and physiological changes. Primary outcomes include physical performance (SPPB), muscle strength (1RM and peak power), and skeletal muscle mass (DXA). Secondary outcomes will evaluate mental health indicators such as fatigue (FACIT-F), resilience, anxiety, depression, and quality of life.

**Discussion:**

This study will provide a detailed framework for implementing ICT-based personalized exercise interventions that incorporate genomic analysis. The EXESALUS program is expected to highlight the potential of tailored high-intensity exercise as a preventive and therapeutic strategy for cancer patients and individuals at risk of chronic diseases. The findings of this protocol will contribute to the development of precision medicine approaches for cancer prevention and management, emphasizing the scalability and utility of ICT-based solutions in health promotion.

**Trial registration:**

This study was registered in the Korean Clinical Trials Registry (KCT0010187).

## Introduction

Cancer is one of the leading causes of death worldwide. Each year, millions of people are diagnosed with cancer, of which many eventually die. In 2020, global cancer incidence estimates showed that there were approximately 19.6 million new cases and 10 million deaths [1]. This mortality rate varies greatly by cancer type, with lung, liver, and stomach cancers having high mortality rates, while survival rates can vary depending on early detection and access to treatment. Many side effects of cancer have been reported, including cognitive decline, depression, fatigue, mortality, sleep disorders, pain, and muscle loss due to cancer cachexia [2]. Additionally, the incidence of cancer varies greatly with age, with certain cancers such as neuroblastoma and acute lymphocytic leukemia (ALL) occurring rarely in children and lung, colorectal, and breast cancers increasing with age, especially in adults over the age of 50. The accumulation of genetic mutations and their close association with cellular aging are the probable causes.

While cancer is devastating to survival and quality of life, exercise has been shown to have both positive and negative effects. However, there are some cancers for which there are no reports on the effects of exercise. Many studies have reported positive effects of exercise (e.g., muscle strength [3], cardiorespiratory fitness, sleep, fatigue, and quality of life [4]) in patients diagnosed with various cancers, including breast and colorectal cancers. Recently, there have been more reports about the positive effects than the negative, such as improving chemotherapy by improving mood fitness. Additionally, the American College of Sports Medicine (ACSM) has also recognized the effectiveness of exercise on cancer and provided guidelines, which has resulted in increased survival rates [5]. The important times to apply exercise interventions are before cancer diagnosis (healthy), during treatment after cancer diagnosis, and survivorship after treatment ends. However, exercise interventions to maintain and promote health and, by extension, cancer prevention, are most important to begin before disease onset. In this study, we will use the identification of genetic variants that are objective indicators of disease causation to guide motivation and implementation of exercise interventions.

Since roughly 2020, the evidence for physical activity and exercise in cancer patients has shifted from negative to positive. Furthermore, the immune effect of killer T cells on CD8 + cells is relatively higher in high-intensity exercise, which reduces fatigue in breast cancer patients [6]; it also inhibits cancer growth by inducing CD8 + cell metabolism [7–9]. In addition, it plays a major role in boosting immunity by increasing natural killer (NK) cell numbers and activity [10,11]. Some chronic diseases (such as pancreatic cancer) are difficult to treat after disease detection, but it is believed that exercise during treatment can improve physical health and quality of life, although more precise exercise programs are needed [12].

Most genomic analysis services are single nucleotide polymorphism (SNP)-based, which means that they predict outcomes using a subset of all genes, thus limiting their predictive accuracy [13]. The occurrence of diseases is due to the multi-layered interaction of several factors, such as hormonal imbalance, environmental factors, and lifestyle, in addition to genes that are congenitally determined. Genetic information alone cannot clearly explain disease onset. With the development of wearable devices and big data analysis technologies, it has become possible to collect and analyze vast amounts of lifestyle and environmental data, which, when combined with personal genomic information, can be used for disease prevention and health promotion to improve quality of life [14]. By accurately predicting an individual’s health risk, suggesting appropriate management plans, and connecting them with health coaching services through the application of appropriate exercise methods, it may become possible to prevent chronic diseases, including cancer, or delay their onset long enough to minimize economic losses for individuals and society.

The current healthcare paradigm is rapidly changing from the existing post-onset treatment method to a prevention-oriented, personalized precision medicine method, and convergence healthcare services using information and communication technology (ICT) have become possible due to the accelerated development of ICT such as mobile networks and applications, Internet of Things, big data, and cloud computing. Therefore, this study aims to identify individual genomic variants, predict cancer prevalence through genomic analysis, and provide an exercise intervention program including high-intensity exercise as a mobile web application to execute personalized exercise, analyze cancer development inhibitory factor expression, and induce muscle strength improvement. Specifically, the purpose of this study is to check the suppressive effect of chronic disease-causing factors, including cancer, based on exercise habits using a mobile web application to predict health risk and establish health-promoting protocols using individual genomic information data.

## Materials and methods

### Study design

This study is a protocol that aims to analyze an individual’s genomic information to predict future health risks and evaluate the health-promoting effects of a personalized exercise program (EXESALUS) based on the results. Additionally, a systematic evaluation of cancer suppressor factor expression, changes in physical strength, and mental health status through a personalized exercise intervention will be conducted.

Participants will follow the EXESALUS exercise program (low-, moderate-, and high-intensity exercise, exercise counseling and guidance, and body information via ICT and wearable devices) for 3 months. Subjects will provide biospecimens (blood, urine, and oral epithelial cells) three times (the beginning of the study, 1 ½ months, and 3 months) to assess genomic information and physiological changes. All biospecimens will be anonymized in accordance with our privacy policy and used for research related to changes in risk markers for chronic diseases, including cancer.

**Fig 1:**
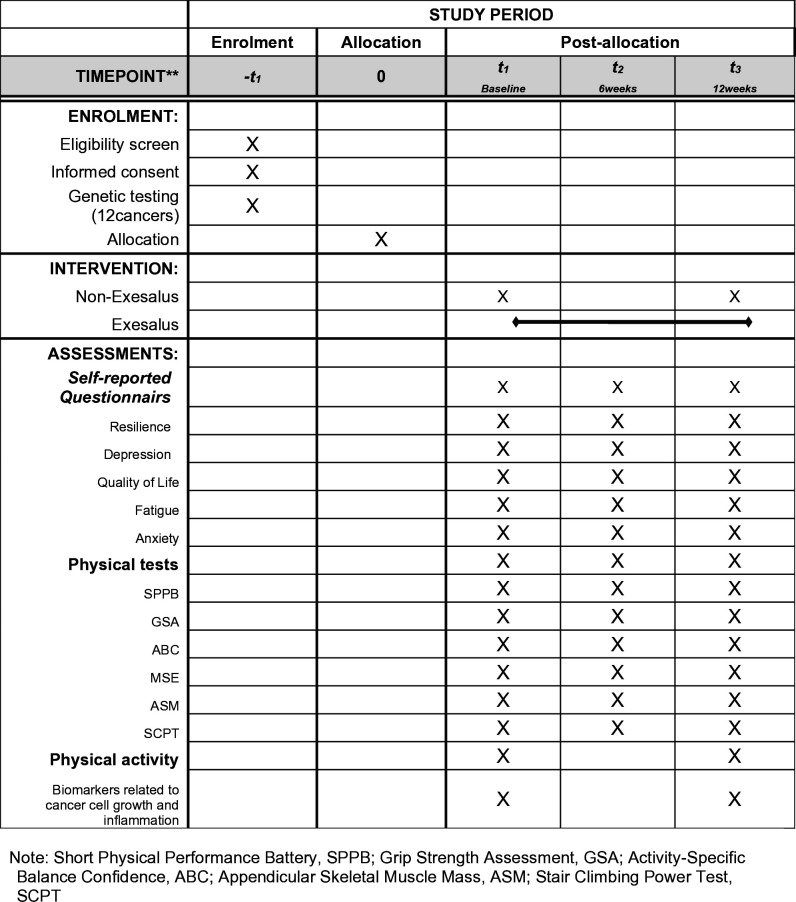
SPIRIT-Figure.

In accordance with the SPIRIT 2013 Checklist.: Recommendations for interventional trial protocol items. (S1 Checklist in S1 File), a Fig 1 is provided to illustrate the time schedule for mental health, physical assessment, and physiological indicator evaluations, along with participant involvement in the study (Fig 1).

### Ethical approval and trial registration

The Institutional Review Board of Yeungnam University granted ethical approval for this study, which has been registered with the Clinical Research Information Service (CRIS), linked to the WHO International Clinical Trials Registry Platform (ICTRP) (KCT0010187). The research data from this clinical study can be assessed on the CRIS platform. (https://cris.nih.go.kr/cris/index/index.do)

To assess mental health, we will use the following scales:

- **Resilience**: Wagnild & Young (1993) [15]
- **Anxiety**: Generalized Anxiety Disorder 7-item Test (GAD-7) [16]
- **Depression**: Brief Edinburgh Depression Scale (K-BEDS) [17]
- **Quality of Life**: McGill Quality of Life Scale (MQOL-K) [18]
- **Fatigue**: Functional Assessment of Chronic Illness Therapy – Fatigue (FACIT-F) questionnaire [19]; the Korean version will be used with prior approval.

This study aims to present new scientific mechanisms that can contribute to physical and mental health promotion and cancer prevention through customized exercise programs and genome-based approaches.

### Subject selection and consent process

#### Recruitment and publicity.

After IRB approval, recruitment will be conducted at all times through posters and promotional materials to recruit subjects on campus and in the surrounding area, including the Yeungnam University Medical Center and Sports Center (S2, S3, S4, S5 in S1 File). Subjects will be included in the study only if they meet the inclusion criteria and voluntarily agree to participate in the study. This study has been approved by the IRB of Yeungnam University (approval number: 7002016-A-2024–069). This study was registered in the Korean Clinical Trials Registry (KCT0010187) on 02/07/2025.

This study is currently ongoing from its approval date, April 16, 2024, to February 28, 2026, as specified in the IRB-approved study plan (S6 in S1 File). The recruitment of participants for this study is planned to be completed by February 28, 2026. Data collection will take place for one year following the completion of participant recruitment, until February 2027. The results of the analyzed data collection are anticipated to occur over the subsequent year, by February 2028. Depending on the number of participants with a history of cancer, the completion timeline may be extended by approximately one year.

The IRB Review Notification (S7 in S1 File) has been included in the supplementary data. Additionally, the confirmation of the independent peer review administered by the funder (S8 in S1 File) has also been provided.

#### Consent to participate in the study.

Prior to the start of the study, the principal investigator and co-investigators will provide the subject with a written and verbal explanation of the study’s purpose and procedures, the privacy and confidentiality of the research materials, the willingness to participate voluntarily, and the potential benefits and risks of the study (S9 in S1 File). After ensuring that subjects fully understand their participation in the research, the study will only proceed if potential participants voluntarily provide written informed consent.

#### Procedure for participation and post-consent.

Consenting subjects will be interviewed and surveyed at a designated location (medical clinic, exercise center, or Yeungnam University Exercise Physiology Laboratory), which will take approximately 1 hour (S10, S11 in S1 File). A suitable time will be arranged with participants.

### Exclusion criteria

Participants will be excluded from the study based on the following criteria:

- Subjects who do not voluntarily agree to participate in the study.
- Subjects who are unable to participate in the exercise program due to physical limitations.
- Elderly people over 80 years of age or children and adolescents under 20 years of age.
- Participating researchers who are likely to be disadvantaged by their superiors due to hierarchy within the organization.
- Subjects who are participating in other studies with similar topics to this study.

To ensure the voluntary participation of the subjects and the fairness of the research process, the above criteria are set to strengthen the objectivity and ethics of the research.

### Subject selection criteria and methods

Subjects will be selected based on the following criteria:

- 300 registered participants of the Health and Exercise Center who participate in exercise at least once a week and voluntarily agree to participate in the study.
- 100 patients registered at Yeungnam University Medical Center.
- 100 members of the general public who do not participate in exercise.

The total number of subjects is set at 500 for the 3-month study period, and the study will be conducted on subjects who agree to participate after being provided information about the study. Written informed consent will be obtained after the exercise specialist confirms that the subject meets the requirements for participation.

### Rationale for subject selection

In this study, we aim to have a sufficient sample size to compensate for the limitation of our previous study [20], which did not yield significant results due to a small sample size (N = 63) while exploring candidate genes through genome-wide association studies (GWAS). To this end, we plan to recruit at least 500 subjects by setting the power (1-β) at 0.8, the effect size at 0.5, and the significance level (α) at 0.05 using the G-power program.

Additionally, after accounting for non-respondents and voluntary/dropouts, the final study population is expected to be around 400. This sample size will increase the statistical significance of the study and contribute to strengthening the reliability of the findings.

### How will samples be collected?

#### Sample type.

Participants will provide a sample of either urine, oral epithelial cells, or blood on three occasions. The total amount of samples collected will be limited to approximately 15 mL of blood or 300 mL of urine. For oral epithelial cells, we will offer multiple choices based on the participant’s request, and blood will only be collected at the hospital for patients enrolled at Yeungnam University Hospital.

- **Oral epithelial cell collection**: A buccal swab will be used to collect oral epithelial cells, and the collection kit will be sent by mail, collected, and referred to a specialized analysis company.
- **Blood collection**: Subjects will have blood drawn from a vein after an 8-hour fast, and the collected blood and urine will be stored in a −80°C ultra-low temperature freezer in the exercise physiology laboratory at Yeungnam University for the duration of the study before being sent to an analytical laboratory.

#### Preservation and disposal of human remains.

All human remains will be preserved and disposed of in accordance with Article 39 of the Act on Bioethics and Safety.

Upon withdrawal of consent to participate in the study or termination of the study, all stored human remains will be classified as medical waste and incinerated, and records related to the disposal (date, amount, method of disposal, etc.) will be kept for 5 years from the date of disposal.

### Serum Analysis (Serum)

- Blood collected from cancer patients will be clotted for 30 minutes at room temperature, centrifuged at 1000 × g for 20 minutes to separate the serum only, and stored at −80°C.
- The serum will be used to measure the expression of factors associated with cancer cell growth (Trim63, Fos, Col1a, and Six2) that have been identified in cell experiments in our laboratory [21]. The cancer development-related markers Human Trim63 (HUF105955; Assay Genie, Dublin, Ireland), Human FOS (HUF101186; Assay Genie), Human Pro-Col1a1 (R&D Systems, Minneapolis, MN, USA), and Human Six2 (ABIN6232377; Antibodies Online, Limerick, PA, USA) will be analyzed using ELISA kits. Additionally, blood will be collected to assess inflammation levels using the Human C-Reactive Protein/CRP Immunoassay (SCRP00B; R&D Systems).

1. Randomization and exercise intervention adjustment

**Fig 2:**
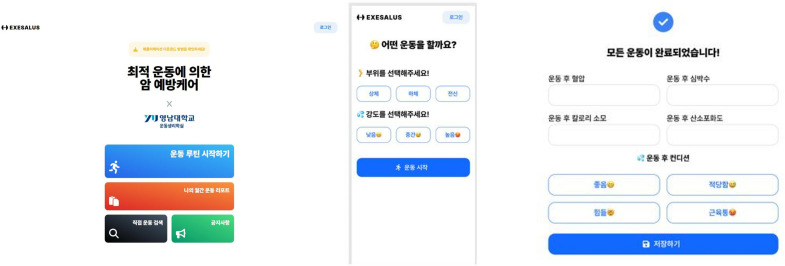
A study-specific app.

After enrollment in the study, all participants will be enrolled in a study-specific app (Fig 2), where they will enter baseline fitness measurements and heart rate data. Based on this, an exercise specialist will adjust the exercise intensity for each subject to provide the appropriate intervention.

**Fig 3:**
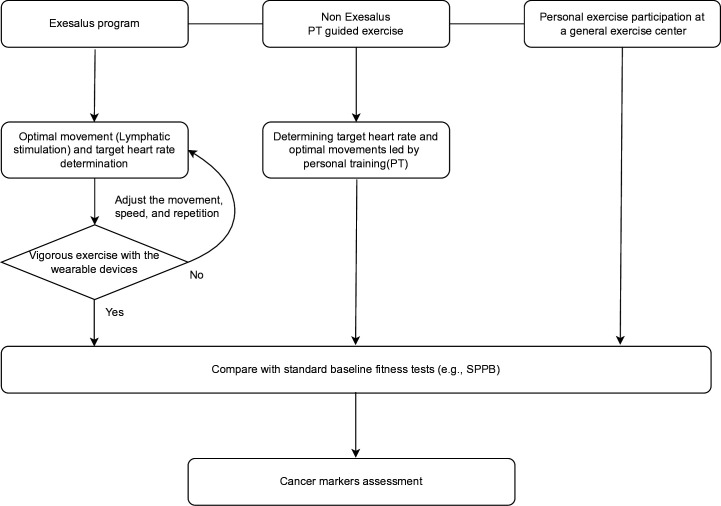
Diagram algorithme.

In this study, the information provided to the exercise intervention group and control group will be different and blinded to ensure that the information is balanced. The exercise intervention group will be provided with a structured exercise program, and they will adjust their own exercise intensity based on their training (Fig 3).

To evaluate the effectiveness of the exercise intervention, all participants will be measured three times. Data will be de-identified with a numeric code per subject and managed so that only the principal investigator will have access to all participants’ identifying information.

2 Exercise interventions

The study will be a 3-month, parallel-group, randomized controlled trial consisting of cancer patients (100) and non-cancer patients (300). The cancer patient group will consist of patients undergoing treatment or post-operative rehabilitation, while the non-cancer patient group will include subjects at more than twice the risk of developing the disease, members of exercise centers, and those who only use ICT apps.

The cancer patient group will be provided with a tailored exercise program to improve cardiorespiratory fitness and muscle strength, which will aim to improve physical function and quality of life.

The **EXESALUS program** is designed so that participants start the exercise intervention within 10 days of enrollment, once their health risk results are confirmed through genetic testing.

If a subject is unable to start the exercise intervention, it will be recorded as a ‘protocol intervention withdrawal.’

Psychological counseling by a clinical psychologist will be provided twice, once before starting the exercise intervention and again at 2-months, to ensure study participation and ongoing motivation.

3-1 EXESALUS Exercise Program

**Fig 4:**
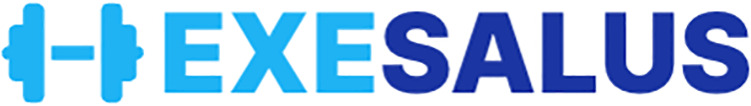
EXESALUS icon.

The EXESALUS exercise program (http://exesalus.com) is an ICT-based personalized exercise intervention program for health promotion and disease prevention (Fig 4). The program provides a variety of exercises targeting the upper body, lower body, abdomen, and whole body (adjusted by intensity) and provides five workouts per week for 3 months, ranging from low to high intensity, using exercises that are appropriate for the target audience. The goal is to build muscle strength and improve psychological well-being.

**Fig 5:**
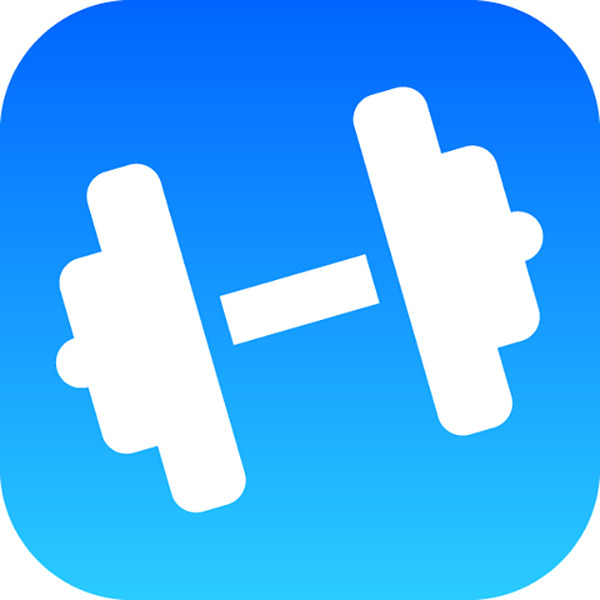
Launcher icon.

**Fig 6:**
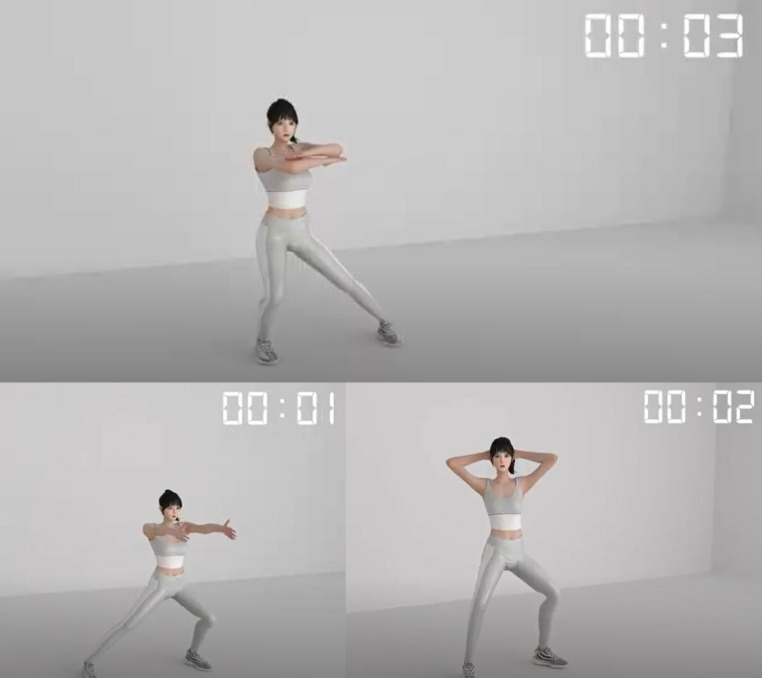
Exercise motions.

The EXESALUS app, designed to help prevent cancer and recurrence, slow muscle loss, and improve physical function, is accessible on mobile or PC (Fig 5) and features 3D customizable animations (Fig 6) and personalized avatars.

The exercise program begins with a light-intensity warm-up session that focuses on flexibility and includes balance exercises customized for each participant. The program consists of warm-up, stretching, and cool-down sessions, and utilizes smartwatches to provide real-time biometric feedback. Unlike traditional exercise programs, EXESALUS has a unique advantage in that it provides high-intensity exercise that is effective in stimulating lymph nodes and lymph glands in cancer prevention and management.

Studies have shown that as physical health improves, cognitive and psychological functioning also shows positive changes. However, the optimal level of exercise intensity and frequency is still being researched. EXESALUS offers a program that consistently delivers low-, medium-, and high-intensity workouts based on proven benefits. Under the guidance of an exercise physiologist without financial burden for 3 months, subjects are guided to perform high-intensity exercise through a customized program (figure diagram algorithm).

3-2 Control group

The non-exercise group will consist of subjects whose risk of developing the disease is 2 or less according to the genetic test.

At the beginning of the intervention, wearable devices and setup assistance will be provided, and the wearable devices will be distributed to 100 subjects.

During the study period, the control group will maintain their daily routine without any specific exercise intervention while self-monitoring using the provided wearable devices. This will allow us to observe and compare changes in the health status of the control group.

### Procedure

#### Data collection and individual counseling.

Study subjects will be monitored for motor awareness (Rate of Perceived Exertion [RPE] of 14 at lactate threshold) through individual counseling, and all voluntarily consented data will be collected, including responses to the app-embedded questionnaire, interview data, and blood samples. All voluntary consenters will be registered in the provided app.

#### Pre-preparation for the exercise program.

Before starting the exercise program, subjects will be informed and prepared through a professional consultation with an exercise physiologist about exercise awareness.

#### Exercise intensity.

The exercise intensity will be set to reach the lactate threshold based on an RPE of 14.

#### Counseling schedule.

Individual counseling sessions will be held at the beginning of the study, after 1½ months, and at the end of 3 months.

### Assessment

Primary outcome measures:

- **Short physical performance battery (SPPB**): A physical activity measurement tool that provides a simple and reliable assessment of a subject’s physical function

Grip strength assessment:

- Measures handgrip strength to assess hand and arm muscle strength

Activity-specific balance confidence (ABC):

- Uses the ABC scale to assess a subject’s confidence in balance.

Muscle strength evaluation:

- Assesses muscle strength levels by measuring maximum strength and peak power.

Appendicular skeletal muscle mass (ASM):

- Measures the amount of skeletal muscle mass in a subject through body composition analysis using Dual Energy X-ray Absorptiometry (DXA).

One repetition maximum strength (1RM) and stair climbing power test:

- Measures 1-rep max strength (1RM) and stair climbing power to assess a subject’s strength and power capacity.

### Privacy protections

This study will utilize data collected from the time IRB approval is received until the end of the study. All personal information collected from study participants will be processed into numerical codes and converted into de-identified secondary data, which will be securely stored and analyzed on the principal investigator’s computer and external hard drive.

The collected personal information (name, gender, date of birth, affiliation, contact information, email, address, past medical history, and medication status) will be used only during the study period and will be kept for 3 years after the study ends, then destroyed in accordance with Article 15 (2) of the Enforcement Rules of the Act on Bioethics and Safety.

The results of the study will only be reported in the form of aggregated data, not individualized information, and if a participant drops out of the study, the data will be immediately deleted from the principal investigator’s external hard drive and the related documents will be destroyed through a shredder. After the end of the study, all study-related data (including data on suspension and withdrawal) will be stored for 3 years in a secure cabinet in the archive located in Cheonma Gymnasium Room 103, after which the information will be permanently deleted. If personal information is leaked, we will compensate for damages in accordance with relevant legal procedures.

### Statistics

The statistical analysis of this study will be centered on the t-test and analysis of variance (ANOVA).

The study population will be divided into two groups: those who lead an active lifestyle and those who do not, reflecting the fact that different exercise centers run different proprietary exercise programs.

Independent variables:

- Active or not active.

Dependent variables:

- Changes in exercise lifestyle (e.g., changes in metrics validated by the exercise physiology lab, gene-related metrics established by Theragen, weight, BMI, etc.)

This will allow us to systematically analyze the impact of exercise life on health metrics and genetic variation.

### Treatment of missing data

The best way to deal with missing data is to plan your research well and collect data carefully to avoid problems. Identify the cause of the omission to minimize the amount of missing data in clinical studies. In this study, if the missing data followed Missing-at Random (MAR), a sensitivity analysis was performed to minimize bias and draw valid conclusions. If the data was MCAR (Missing Completely at Random), the data was deleted by Listwise or case deletion and the data were evaluated [22].

## Discussion

In this study, we will evaluate the impact of personalized exercise intervention on the physical and mental health of cancer patients through the EXESALUS mobile application. The exercise program of EXESALUS is aimed at high-intensity exercise through the animation of 3D avatars to make exercise fun and sustainable. Currently, wearable devices such as smartbands and smartwatches that measure users’ physical activity are available as tools to support this activity; however, wearable devices alone do not make exercise interesting, and it is difficult to perform high-intensity exercise customized to the user.

For people with cancer, research shows that maintaining high-intensity physical activity is necessary. Evidence suggests that CD8+ and NK cells of the immune system, which play an important role in the defense against cancer cells and are activated during exercise, are specifically and significantly activated at high intensity (more so than CD4 + cells) [23–25].

This study is different from existing exercise programs in that it consists of movements designed to increase the heart rate due to high intensity, stimulate lymph nodes and lymph glands, activate related cells (such as T cells) in the lymph that are responsible for immunity, and promote lymphocyte circulation in the systemic lymphatic vessels. Above all, it provides feedback that collects and graphs biometric information to help users apply the right intensity and exercise to their bodies. We planned this study in collaboration with a gene analysis company to anticipate the onset of cancer through gene-based analysis and apply high-intensity preventive exercises to inhibit cancer development.

The results of the study will confirm that a tailored high-intensity exercise program has significant effects on improving physical function, reducing fatigue, and enhancing immune function in cancer patients, which will support the positive effects of exercise on cancer-related health improvements reported in previous studies [26].

The application of the EXESALUS app in this study may also demonstrate the potential to expand the accessibility of exercise interventions to cancer patients, particularly those living in rural and remote areas. Upgrading the current mobile app with features such as real-time mirroring and postural feedback would allow users to perform exercises independently and provide personalized exercise instructions. These remote support features could be useful as an accessible exercise intervention in healthcare resource-poor areas [27].

Additionally, the EXESALUS program could emphasize community support to better engage cancer patients in exercise interventions. Consistent with existing research, social support is an important factor for cancer patients to engage consistently in exercise interventions [28]. Consequently, the EXESALUS program is designed to motivate participants by combining personalized instruction with community-based group interaction, which will lay the foundation for sustained participation in the program over time.

The positive effects of tailored exercise interventions on reducing cancer-related fatigue and improving quality of life are well documented [29] and will be confirmed in this study. The tailored approach will meet the specific needs of the patients and will contribute to minimizing fatigue while promoting mental well-being. These results will highlight the need to develop tailored exercise prescriptions optimized for cancer patients.

In addition, the EXESALUS mobile application will be able to show long-term applicability across all phases of cancer treatment (pre-diagnosis, during treatment, and survivorship). It will suggest that digitally-based personalized exercise programs can improve physical recovery and quality of life in the long term, and it will increase the scalability of ICT-based health solutions in chronic disease management [30–31].

### Future research directions

This study will support that personalized app-based exercise programs have a positive impact on improving the health of cancer patients. In particular, the EXESALUS program is a scalable and accessible exercise intervention model that has the potential to be utilized at various stages of cancer treatment. Based on the user’s medical history and biometric information gathered from the program, we are also considering the possibility of developing customized supplements for cancer cachexia-induced sarcopenia.

The continued development of this model will have a significant impact on cancer management and could improve patient survival and quality of life through personalized exercise strategies.

## Supporting information

S1 File S1 SPIRIT checklist. S2 Recruitment of research participants. S3 Yeungnam University Research Participant Recruitment Poster. S4 Leaflet Brochure. S5 3 banners. S6 the study plan translator. S7 IRB Review Notification translator. S8 the funding certification. S9 Human Subjects Research Consent Explanation and Consent Form. S10 Medical history questionnaire. S11 Exercise participation questionnaire.
(ZIP)
