# Supplementary material for: The protocol for developing health and disease prevention services: An exercise-based prediction model integrating genomic test results
Source: PLoS One. 2025 Jul 22;20(7):e0327947. doi: 10.1371/journal.pone.0327947 (PMC12282888; doi:10.1371/journal.pone.0327947)
Supplement: S1 File — S1 SPIRIT checklist. S2 Recruitment of research participants. S3 Yeungnam University Research Participant Recruitment Poster. S4 Leaflet Brochure. S5 3 banners. S6 the study plan translator. S7 IRB Review Notification translator. S8 the funding certification. S9 Human Subjects Research Consent Explanation and Consent Form. S10 Medical history questionnaire. S11 Exercise participation questionnaire. (ZIP) [file pone.0327947.s001.zip › S10 Medical history questionnaire.pdf]

Yeungnam University Exercise Physiology Department
Physical Test Manual

Part 1. Medical history questionnaire: completed by the participant himself/herself

Part 1: 병력 질문지

date:____________ **Last created on:**__________

---

name:______________________Gender: Male / Female Date of Birth:_________________________age:_______________

Affiliation:______________________contact:___________________email:_____________________

address:____

____

Emergency contact information:_________________relationship:_________name:___________________(person)

Instructions. The questions below are about medical history. Please read carefully and circle the items that apply. If the answer is " yes " , please provide details in the space provided below.

1. Do you have the following experience or are you currently in the following condition?

go. Have you ever experienced limitations in exercising beyond your health?
Yes/No/Don't know

me. Have you recently had a new illness or injury? **Yes/No/Don't know**

all. Are you suffering from a chronic illness or a specific illness? (e.g. diabetes, asthma, etc.)
Yes/No/Don't know

la. Have you ever had an allergic reaction to bees, pollen, latex, or food?
Yes/No/Don't know

mind. Do you have a blood condition such as anemia, hemophilia, or sickle cell anemia?
Yes/No/Don't know

bar. Did you have a family member who died before the age of 50? If so, please describe in detail below.
Yes/No/Don't know

Please describe in detail (and include the date) the items you answered " yes " to below .

____

____

____

____

____

____

Please list below the medications you have recently taken or are taking.

drug name	dosage	number

2 . Have you experienced or currently have any of the following heart-related diseases?

go. Have heart problems ever limited your ability to participate in exercise?

Yes/No/Don't know

me. Have you ever experienced chest pain or discomfort?

Yes/No/Don't know

all. Have you ever experienced a heart murmur (heart murmur)?

Yes/No/Don't know

la. Have you ever experienced high blood pressure?

Yes/No/Don't know

mind. Have you experienced elevated cholesterol levels? **Yes/No/Don't know**

bar. Have you ever experienced dizziness during or after exercise for no known reason?

Yes/No/Don't know

buy. Have you ever had a cardiac stress test done by your doctor?

Yes/No/Don't know

(e.g., extensibility , echocardiography, exercise stress testing, and ambulatory electrocardiography)

ah. Have you ever experienced unexplained shortness of breath or sudden fatigue while exercising?

Yes/No/Don't know

car. Does anyone in your family have the following medical history?

Yes/No/Don't know

(1) Those who experienced heart disease under the age of 50

Yes/No/Don’t know

(2) Those who died without any specific reason

Yes/No/Don't know

(3) Person who died during exercise

Yes/No/Don’t know

3 . Have you ever experienced any of the following general or exercise-related abnormal signs or symptoms?

go. Have you ever experienced difficulty breathing?

Yes/No/Don't know

(1) During exercise

Yes/No/Don’t know

(2) After running 1 km **Yes/No/Don’t know**

(3) Have you ever experienced coughing, wheezing, or difficulty breathing due to seasonal changes?

Yes/No/Don't know

(4) Have you ever experienced exercise-induced bronchoconstriction?

Yes/No/Don't know

i. Have you ever experienced dizziness or fainting?

Yes/No/Don't know

me. Have you ever had a viral infection (infectious mononucleosis, hepatitis, or infectious cold)? **Yes/No/Don't know**

all. Do you tire more easily than other people? **Yes/No/Don't know**

" Please provide a detailed explanation of the items you answered yes to, including dates.

---

4 . Have you been exercising regularly in the past month ? yes/no

" For the items you answered 'no' to, if you have exercised in the past 1 month, please explain in detail what kind of exercise you did and whether you exercised regularly.

" For items where you answered yes, please explain in detail what type of exercise (aerobic, anaerobic, etc.) you performed on a regular basis.
