# Supplementary material for: The protocol for developing health and disease prevention services: An exercise-based prediction model integrating genomic test results
Source: PLoS One. 2025 Jul 22;20(7):e0327947. doi: 10.1371/journal.pone.0327947 (PMC12282888; doi:10.1371/journal.pone.0327947)
Supplement: S1 File — S1 SPIRIT checklist. S2 Recruitment of research participants. S3 Yeungnam University Research Participant Recruitment Poster. S4 Leaflet Brochure. S5 3 banners. S6 the study plan translator. S7 IRB Review Notification translator. S8 the funding certification. S9 Human Subjects Research Consent Explanation and Consent Form. S10 Medical history questionnaire. S11 Exercise participation questionnaire. (ZIP) [file pone.0327947.s001.zip › S11 Exercise participation eligibility screening.pdf]

영남대학교 운동 생리학실
Yeungnam University exercise physiology lab

PAR-Q&YOU

(Exercise participation eligibility screening)

This questionnaire is the minimum standard that can be used as a reference when entering physical activity. PAR-Q indicates exercise aptitude , and depending on the results, it may be inappropriate to exercise excessively or the exercise is most suitable for you. You can find out who needs a medical opinion as to what it is. These days, the number of people who exercise for health reasons is increasing. For most people, there is no harm in exercising a lot, but some people may need to see a doctor before starting an exercise program. Therefore, before starting a physical activity program, please answer the seven questions below: I will do it.

	**yes**	**no**
ever been told by your doctor that you have heart disease and should only exercise as recommended by your doctor? Are there any?	☐	☐
2. Have you ever felt pain in your chest when exercising?	☐	☐
3. Have you recently felt pain in your chest even when you are still?	☐	☐
4. You dizziness because balance bereaved enemy Do you have it ? or Even once consciousness bereaved enemy Do you have it ?	☐	☐
5. Is it likely that your bone or joint condition will worsen if you exercise?	☐	☐
6. Have you ever been prescribed medication for blood pressure or heart disease by your doctor?	☐	☐
7. Are there any other reasons why we should not exercise too hard?	☐	☐

yes" to one or more of these questions, it is recommended that you consult your doctor before starting or increasing the amount of exercise you do. Anyone who can answer " no" to all of the above questions can safely consider starting an exercise program . If your health condition changes while participating in an exercise program and you need to answer " yes" to any of the seven questions above, it is best to consult a doctor. great.

◉ Read the seven questions above and I understand.

name :________________signature :___
