# Supplementary material for: The protocol for developing health and disease prevention services: An exercise-based prediction model integrating genomic test results
Source: PLoS One. 2025 Jul 22;20(7):e0327947. doi: 10.1371/journal.pone.0327947 (PMC12282888; doi:10.1371/journal.pone.0327947)

# Recruitment of research participants

**Research title: Effect of exercise lifestyle in establishing a prediction model for health improvement through exercise according to genome test results and development of health and disease prevention management services based on this**

1. Purpose of the study:

   We aim to determine health promotion's impact by knowing health risks based on personal genetic information and adding appropriate exercise interventions to daily life.

2. Criteria for selecting subjects:

   Registered participants at the Health Exercise Center and registered patients at Yeungnam University Medical Center

3. Research Location and Period: Yeungnam University Exercise Physiology Department (Room 103, Cheonma Gymnasium, 280, Daehak-ro, Gyeongsan-si) and Medical Center (170 Hyeonchung-ro, Nam-gu, Daegu Metropolitan City)

   BM Co.,Ltd. (150-3 Seokchon-dong, Songpa-gu, Seoul),

   Chris Workout (194 Hayang-ro, Hayang-eup, Gyeongsan-si)

   Permanent recruitment form January 2024

4. Contents and Duration:

   Blood(5ml), Urine(100ml), or a small amount of oral mucosa using a cotton swab were collected three times at each time point, at the start of the study, at one and a half months, and after the third month. In the case of exercise intervention, it was performed at each sports center and the app could be used

5. Compensation for research subjects:

   Detailed explanations and related data on genome analysis results that can help prevent individual disease

   Distribution and analysis cost reduction benefits of close to 50%

## Inquiry

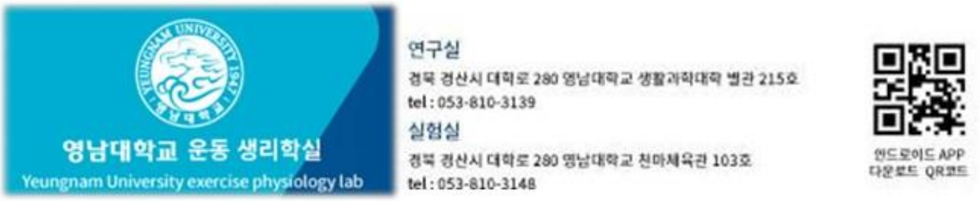

Supplement: S1 File — S1 SPIRIT checklist. S2 Recruitment of research participants. S3 Yeungnam University Research Participant Recruitment Poster. S4 Leaflet Brochure. S5 3 banners. S6 the study plan translator. S7 IRB Review Notification translator. S8 the funding certification. S9 Human Subjects Research Consent Explanation and Consent Form. S10 Medical history questionnaire. S11 Exercise participation questionnaire. (ZIP) [file pone.0327947.s001.zip › S2 Recruitment of research participants.pdf]
