# Supplementary material for: The protocol for developing health and disease prevention services: An exercise-based prediction model integrating genomic test results
Source: PLoS One. 2025 Jul 22;20(7):e0327947. doi: 10.1371/journal.pone.0327947 (PMC12282888; doi:10.1371/journal.pone.0327947)
Supplement: S1 File — S1 SPIRIT checklist. S2 Recruitment of research participants. S3 Yeungnam University Research Participant Recruitment Poster. S4 Leaflet Brochure. S5 3 banners. S6 the study plan translator. S7 IRB Review Notification translator. S8 the funding certification. S9 Human Subjects Research Consent Explanation and Consent Form. S10 Medical history questionnaire. S11 Exercise participation questionnaire. (ZIP) [file pone.0327947.s001.zip › S3 Yeungnam University Reserch Participant Recruitment Poster.pdf]

EXE SALUS

영남대학교 운동 생리학실

연구 참여자 모집

연구제목 : 유전체 검사결과에 따른 건강 증진 운동모델 수립과 효과 검증

연구목적
개인의 유전체 정보를 바탕으로 건강 위험도를 알고 적절한 운동 중재를
지속함으로 건강위험 개선에 미치는 영향을 알아보고자 함

대상자
①운동에 참여하지 않는 일반인
②exesalus앱(http://exesalus.com)을 통해 운동에 참여 하실분
(앱 비활용 참여자도 연구참여가능)
③건강운동센터를 통해 운동에 참여 하실분

연구장소
운동센터, 영남대학교 운동생리학실(경산시 대학로 280 천마체육관 103호), 체육관

내용및소요시간
운동참여, 설문참여, 면봉을 이용한 소량의 구강점막과 타액 채취
운동중재의 참여기간은 최대 3개월 소요될 예정

연구 대상자에 대한 보상
①exesalus 앱 무료 이용
②유전자검사료 70% 혜택(개인부담금 55,000원), 암 환자의 경우 연구검사비 면제
※ 개인별 유전체분석결과에 대한 상세한 설명과 관련자료 배부

문의
E-MAIL
jeehs@ynu.ac.kr

연구실
생활과학대학
별관215호
053.810.3139

실험실
천마체육관 103호
053.810.3148

영남대학교 운동 생리학실
Yeungnam University exercise physiology lab

안드로이드APP다운로드QR코드
