# Supplementary material for: The protocol for developing health and disease prevention services: An exercise-based prediction model integrating genomic test results
Source: PLoS One. 2025 Jul 22;20(7):e0327947. doi: 10.1371/journal.pone.0327947 (PMC12282888; doi:10.1371/journal.pone.0327947)
Supplement: S1 File — S1 SPIRIT checklist. S2 Recruitment of research participants. S3 Yeungnam University Research Participant Recruitment Poster. S4 Leaflet Brochure. S5 3 banners. S6 the study plan translator. S7 IRB Review Notification translator. S8 the funding certification. S9 Human Subjects Research Consent Explanation and Consent Form. S10 Medical history questionnaire. S11 Exercise participation questionnaire. (ZIP) [file pone.0327947.s001.zip › S4 leaflet Brochure.pdf]

'암' 예방 운동케어

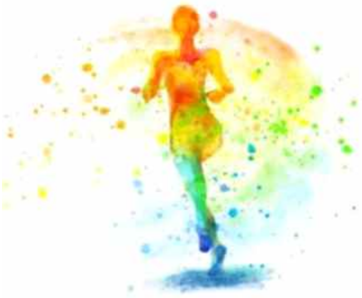

어떤 운동을 해야 할까?

EXESALUS

영남대학교
Yeungnam University

영남대학교 운동 생리학실
Yeungnam University's Center for Exercise Physiology

최적 운동에 의한

암 예방케어

EXESALUS

연구실
경북 경산시 대학로 280 영남대학교 생활과학대학 별관215호
TEL : 053-810-3139 Email : jeehs@ynu.ac.kr

실험실
경북 경산시 대학로 280 영남대학교 천마체육관 103호
TEL 053-810-3148 Email : jeehs@ynu.ac.kr

암 예방의 근거는?

협력업체의
유전체 예측 분석으로
해당 암의 발생위험도 검사

운동의 종류, 강도, 빈도,
시간 등의 조절로
개인 맞춤형 운동자극에
의한 신체의 특징 암 대응의
체제유도

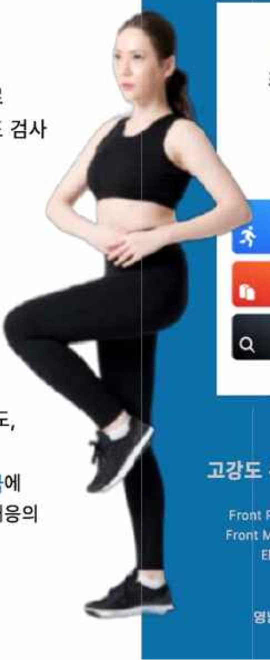

'암' 예방을 위한 트레이닝

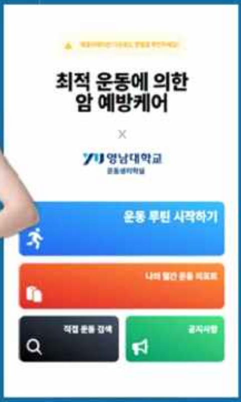

근저 중심의
고강도 유산소 운동 프로그램

Front Physiol, 2023, 13:1078512
Front Mol Biosci, 2022, 9:818470
Elife, 2020, 9:e59996

영남대학교 운동생리학실

근거 중심의 운동, 동작 예시

EXESALUS의 운동동작일부

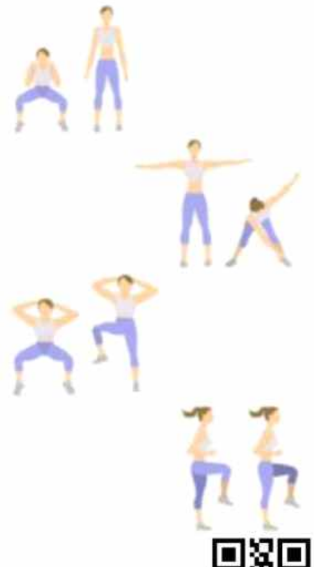

안드로이드 플레이스토어에서
'암' 예방운동 검색
