# Supplementary material for: The protocol for developing health and disease prevention services: An exercise-based prediction model integrating genomic test results
Source: PLoS One. 2025 Jul 22;20(7):e0327947. doi: 10.1371/journal.pone.0327947 (PMC12282888; doi:10.1371/journal.pone.0327947)
Supplement: S1 File — S1 SPIRIT checklist. S2 Recruitment of research participants. S3 Yeungnam University Research Participant Recruitment Poster. S4 Leaflet Brochure. S5 3 banners. S6 the study plan translator. S7 IRB Review Notification translator. S8 the funding certification. S9 Human Subjects Research Consent Explanation and Consent Form. S10 Medical history questionnaire. S11 Exercise participation questionnaire. (ZIP) [file pone.0327947.s001.zip › S5 3 banners.pdf]

EXESALUS

유전자 건강 체크로
삶의 질을높 이세요!

근거 기반의
효율적인 운동만이
당신의 삶의 질을
향상시킬 수 있습니다

자세한 내용은 팜플렛(연구참여자 모집문건)을
참조하세요

바로가기 》

문 의

영남대학교운동생리학실
Yeungnam University School of Exercise Physiology

TEL 053-810-3139
jeehs@ynu.ac.kr

YU 영남대학교 운동생리학실

THERAGEN

EXESALUS

유전자 건강 체크로
삶의 질을높 이세요!

근거 기반의
효율적인 운동만이
당신의 삶의 질을
향상시킬 수 있습니다

자세한 내용은 팜플렛(연구참여자 모집문건)을
참조하세요

바로가기 》

문 의

영남대학교운동생리학실
Yeungnam University School of Exercise Physiology

TEL 053-810-3139
jeehs@ynu.ac.kr

YU 영남대학교
운동생리학실

THERAGEN

EXESALUS

유전자 건강 체크로
삶의 질을높 이세요!

근거 기반의
효율적인 운동만이
당신의 삶의 질을
향상시킬 수 있습니다

자세한 내용은 팜플렛(연구참여자 모집문건)을
참조하세요

바로가기 》

문 의

영남대학교운동생리학실
Yeungnam University School of Exercise Physiology

TEL 053-810-3139
jeehs@ynu.ac.kr

YU 영남대학교 운동생리학실

THERAGEN
