# Supplementary material for: The protocol for developing health and disease prevention services: An exercise-based prediction model integrating genomic test results
Source: PLoS One. 2025 Jul 22;20(7):e0327947. doi: 10.1371/journal.pone.0327947 (PMC12282888; doi:10.1371/journal.pone.0327947)
Supplement: S1 File — S1 SPIRIT checklist. S2 Recruitment of research participants. S3 Yeungnam University Research Participant Recruitment Poster. S4 Leaflet Brochure. S5 3 banners. S6 the study plan translator. S7 IRB Review Notification translator. S8 the funding certification. S9 Human Subjects Research Consent Explanation and Consent Form. S10 Medical history questionnaire. S11 Exercise participation questionnaire. (ZIP) [file pone.0327947.s001.zip › S6 the study plan translator.pdf]

Appendix 2-1 Research Plan (Human Subject Research)

Version1.3

Research Plan (Human Subject Research)

Version: 1.3

1. **Research title**

(국문) 유전체 검사결과에 따른 운동생활에 의한 건강 증진 예측모델 수립에 있어 운동생활습관의 효과와 이를 기반한 건강 및 질환예방 관리 서비스 개발

(영문) Development of health and disease prevention managing services based on the effectiveness of exercise lifestyle in establishing a prediction model for health promotion by exercise based on genomic test results.

2. **Names and positions of the principal investigator and researchers**

	Name	Affiliation (Address)	Position
Pricipal Investigator	Ji Hyun-seok	Yeungnam University, Department of Physical Education	Assistant Professor
Co-researcher	Choi Yun-suk	Yeungnam University, Department of Physical Education	Postgraduate student

3. **Research period**

Institutional Review Board approval date~ February 28, 2026

4. **Research location**

Research Institution:

Exercise Physiology Lab, Yeungnam University (Room 103, Gheonma Gymnasium, 280 Daehak-ro, Gyeongsan-si, Gyeongsangbuk-do)

Terrazen Etex Co., Ltd. (4th floor, Building A, Next-generation Convergence Technology Institute, 145 Gwanggyo-ro, Yeongtong-gu, Suwon-si, Gyeonggi-do)

5. **Research Background**

❍ Health risk analysis based on genetic information has clear limitations in predictive accuracy due to its inherent characteristics. (Hirschhorn et al.,2022)

-Most genome analysis services are SNP-based, predicting results using a portion of the entire genome. Therefore, there is a limit to the prediction accuracy.

-The occurrence of disease is determined by various factors, including hormonal imbalance, environmental factors, and lifestyle habits, in addition to innately determined genes. The disease is caused by layered interactions, and genetic information alone cannot explain the disease.

❍ As wearable devices and big data analysis technology have developed, it has become possible to collect and analyze a vast amount of lifelog and environmental data, and when this is linked to an individual’s genetic information, more accurate health risk predictions are possible.

❍ If the health risk of an individual is accurately predicted, appropriate management measures are suggested, and health management coaching services are linked through the application of appropriate exercise methods, the economic loss to individuals and society can be minimized by sufficiently delaying the onset or prevention of chronic diseases, including cancer.

-Some chronic diseases (such as pancreatic cancer) are difficult to treat after discovery, so preventive management is the best option.

❍ Health care and healthcare that combine big data, artificial intelligence, genome, wearable and IoT technologies are research topics that fit the 4th industrial revolution.

- Thanks to the accelerated development of information and communication technologies such as mobile networks/applications, the Internet of Things, big data, and cloud, centered on ICT, convergence healthcare services utilizing these have become possible.
- The current medical paradigm is rapidly changing from the existing post-onset treatment method to a predictable, preventive, personalized precision medicine method.

6. Research Purpose

The effectiveness of exercise life in establishing a health risk and health promotion prediction model using personal genomic information data and the development of health and disease prevention and management services based on this

-The effect of using exercise-related apps to suppress chronic disease-causing factors, including cancer

-Inhibitory effect of chronic disease-causing factors including cancer according to exercise lifestyle

7. Research Method

This study is an academic study that aims to predict health risks based on an individual's genetic information and to find out the effect of adding appropriate exercise intervention to daily life based on the predicted results on health promotion. It aims to find out how changes in genetic markers indicating various chronic diseases including cancer change through appropriate exercise based on evidence. The human body tissues collected through this study will be stored and managed in a way that personal information cannot be identified, and will be used to evaluate changes in markers indicating chronic diseases including cancer in the future and to understand the mechanisms and conduct genetic research. This study plans to utilize the following methods.

- Consent of research subjects

Before the start of the research, the principal investigator and co-researchers will directly explain to the subjects the purpose and progress of the research, the protection of personal information and confidentiality of research data, the voluntariness of consent to participate in the research, and the benefits and risks. The content will be explained verbally. After confirming the subjects' understanding of the research, the research will proceed only with those who voluntarily sign the consent form.

- Role of research subjects

Research subjects are outpatients of Yeungnam University Medical Center, participants in each exercise program (including app use) registered at the exercise center, and the general public who did not participate in exercise. They participated in the sample collection described below for 3 months.

- Research progress and overview

The research period is 3 months, and for the treatment of human-derived materials, the subjects will select one of the following 3 samples (blood, urine, oral epithelial cells) and proceed with the research 3 times: at the start of the research, one month and a half later, and in the third month.

- Method of collecting samples

Type: urine, oral epithelial cells, or blood, choose one (total of 3 collections, total amount: blood approximately 15 mL, urine 300 mL) (In addition to oral epithelial cells, there were also requests from subjects in the preliminary survey, so the selection was varied. In the case of blood (only for hospital-registered patients), it was collected at Yeungnam University Hospital.

Quantity: Up to 1,500 (the amount collected may be reduced if the subject refuses or withdraws)

Collection and specimen storage location: After the sample collection kit is mailed, collect it, Yeungnam University Exercise Physiology Lab, and each exercise center (in the case of blood or urine), store it in a -80degree Celsius freezer in the Exercise Physiology Lab, and then send it to the analysis company. The remaining samples will be classified as medical waste and incinerated.

Collection method: Buccal swab for oral epithelial cells or blood collection from a vein after 8 hours of fasting

Choose one of the following: blood, urine, oral tissue: Approximately 5 mL of blood, 100 mL of urine, and a small amount of oral mucosa using a cotton swab at the beginning and at 1.5 months and after the third month

Total amount: Approximately 15 mL of blood, 300 mL of urine

- Preservation and disposal of human-derived materials, etc.: Under Article 39 of the Bioethics and Safety Act, when research consent is withdrawn or research is terminated (during the period specified in the consent form), all stored human-derived materials will be classified as medical waste and incinerated, and records (date and time of disposal, amount of disposal, disposal method, etc.) will be kept for 5 years from the date of disposal.

-Providers

Yeongnam University Medical Center (170 Hyeonchung-ro, Nam-gu, Daegu),

BM Co., Ltd. (150-3 Seokchon-dong, Songpa-gu, Seoul),

Chris Workout (194 Hayang-ro, Hayang-eup, Gyeongsan-si)

Observation of changes before and after treatment of various cancer-related indicators (indicators verified by our exercise physiology lab, 12 types of cancer including Trim63, Fos, Col1a1, and Six2)

-Comparison group

General exercise (aerobic and strength training combined exercise) performed at a fitness center for at least a month vs. high-intensity aerobic exercise using an app

Changes before and after exercise by each medical history

Changes before and after exercise by each age group

Since multiple fitness centers were selected, each fitness center will have its own unique exercise program.

Therefore, as in the title, we classified them into groups that exercised as part of their lifestyle and groups that did not. Therefore, the independent variable can be exercise life vs. the dependent variable can be changes due to exercise life (e.g., changes in indicators verified by the exercise physiology lab, indicators set by Teragen, weight BMI, etc.).

- Utilization data

Genomic data: Using Thermofisher's Asian PMRA chip, human-derived materials from research participants provided by the Exercise Physiology Lab and each exercise center at Yeungnam University were analyzed to produce approximately 800,000 SNP information.

In addition, you can fill out a medical history questionnaire and an exercise eligibility test-related questionnaire.

- Statistical analysis method: Analysis will be mainly conducted using t-test and ANOVA.

8. Selection criteria and exclusion criteria for research subjects

Selection criteria: 400 participants registered at the Health and Exercise Center (those who exercise at least once a week)

(A study on 100 patients registered at Yeungnam University Hospital will be conducted by Professor Park Cheol-Hyeon of Yeungnam University Hospital after receiving approval from the IRB of Yeungnam University Hospital.)

- Exclusion criteria:

Those who voluntarily refused to participate, those who were unable to move and thus could not participate in the exercise program.

Elderly people over 80 years of age, or children and adolescents under 20 years of age.

Participating researchers will be excluded from the research subjects because there is concern about disadvantages from superiors in the organizational hierarchy.

9. Expected number of research subjects and basis for calculation.

A previous study that explored candidate gene composition using genome-wide association studies (GWAS) found the results significant.

Because the sample size (N=63) was small due to the lack of this reason (Kim et al., 2022), for this study, we plan to collect data by recruiting subjects based on the minimum number of 500 people calculated using the G-power program with a power (1-β) 0.8, an effect size of 0.5, and a significance level(α) of 0.05. Excluding the 100 or non-respondents and voluntary/dropouts, the minimum actual target population is expected to be around 400.

10. Risks and benefits for research subjects

No adverse effects that may occur in this study have been reported to date. However, none of the subjects experienced any discomfort during participation. If the subject complains of a problem, the experiment can be stopped at any time, and the subject is fully informed that this has no disadvantages. The health

of the participant and the patient before and after exercise can be known at the genetic level, and there is an advantage to improving health by participating in the exercise intervention.

11. Compensation for research subjects

Through this study, detailed explanations of the experimental results and related materials will be distributed to the subjects. This is a type of body screening, and it will be help prevent diseases for each subject by providing an opportunity to find genetic risk factors of the subject and correct them early. In addition, participants will bear the analysis cost of 50,000 won, thereby reducing the total cost.

You will be able to benefit from a reduction in analysis cost of about 100,000 won out of the 150,000 won in research cost. (However, those with cancer experience will be covered by research funds)

12. Method of recruiting research subjects

From the date of IRB approval, we will recruit subjects on an ongoing basis through poster attachments on campus and in the vicinity, including Yeungnam University Medical Center and Sports Center. Among those interested in this study, only those who meet the research subject selection criteria and voluntarily agree to participate in the study will be included. (Astudy on 100 patients registered at Yeungnam University Hospital will be conducted later by Professor Park Chul-hyun of Yeungnam University Hospital will be conducted later by Professor Park Chul-hyun of Yeungnam University Hospital after approval from the IRB of Yeungnam University Hospital.)

13. In case of research involving subjects in vulnerable environments, protective measures for them

Participating researchers will be excluded from the research subjects because they are concerned about being disadvantaged by superiors in the organizational hierarchy.

14. When collecting personal information, what protection measures are taken for it.

This study will use data collected during the period from IRB approval to the end. Personal data collected from subjects through participation in the study will be processed into numbers, converted into secondary data that can be stored as files, and stored and analyzed on an external hard drive in the research director's computer. This personal information (name, gender, date of birth, affiliation, contact information, email, address, medical history, medication use) will be used during the research period, and records and storage of human subject research will be stored for 3 years from the end of the research and then destroyed in accordance with Article 15, Paragraph 2 of the Enforcement Regulations of the Bioethics and Safety Act. The information on the subjects will be described in the research results as whole data form, not as individualized data. If any subject fails all tests and the research is not completed, the data will be completely deleted from the research director's external hard drive immediately and the related documents will be destroyed with a shredder. After the research is completed, research-related data (data on discontinuation of participation and withdrawal will be destroyed) will be stored in the data archive in the laboratory (Room 103, Cheonma Gymnasium) for three years and will be permanently deleted after being stored in a safe-keeping cabinet under the supervision of the research director. If personal information is leaked, damages will be compensated according to legal procedures.

15. References

Kim C, Hong KW, Park DH, Chun S, Oh S, Park Y, Kim K, Choi SW, Jo H. Lung-and liver-dominant phenotypes of Korean eight constitution medicine have different profiles of genotype associated with each organ function. Physiological Reports. 2022 Sep;10(17):e15459.

Hirschhorn JN, Lohmueller K, Byrne E, Hirschhorn K. A comprehensive review of genetic association studies. Genetics in medicine. 2002 Mar;4(2):45-61.
