# Supplementary material for: The protocol for developing health and disease prevention services: An exercise-based prediction model integrating genomic test results
Source: PLoS One. 2025 Jul 22;20(7):e0327947. doi: 10.1371/journal.pone.0327947 (PMC12282888; doi:10.1371/journal.pone.0327947)
Supplement: S1 File — S1 SPIRIT checklist. S2 Recruitment of research participants. S3 Yeungnam University Research Participant Recruitment Poster. S4 Leaflet Brochure. S5 3 banners. S6 the study plan translator. S7 IRB Review Notification translator. S8 the funding certification. S9 Human Subjects Research Consent Explanation and Consent Form. S10 Medical history questionnaire. S11 Exercise participation questionnaire. (ZIP) [file pone.0327947.s001.zip › S7 IRB Review Notification translator.pdf]

Yeungnam University Institutional Review Board
IRB Review Notification

<table>
<tr><td>Project Number</td><td>2023-R-1203-003</td><td>IRB approval Number</td><td colspan="2">7002016-A-2024-069</td></tr>
<tr><td>Research Title</td><td colspan="4">(국문) 유전체 검사결과에 따른 운동생활에 의한 건강 증진 예측모델 수립에 있어 운동생활 습관의 효과와 이를 기반한 건강 및 질환예방 관리 서비스 개발
(영문) Development of health and disease prevention managing services based on the effectiveness of exercise lifestyle in establishing a prediction model for health promotion by exercise based on genomic test results.</td></tr>
<tr><td rowspan="3">Principal Investigator</td><td>Name</td><td>Hyunseok Jee</td><td>Affiliation</td><td>Yeungnam Univetsity/Department of Physical Education</td></tr>
<tr><td>Position</td><td>Professor</td><td>Contact</td><td>010-4900-3213</td></tr>
<tr><td>Email</td><td colspan="3">Jeehs@ynu.ac.kr</td></tr>
<tr><td>Type of Research</td><td colspan="2">☑ Human Subject Research
☑ Human-Derived Materials Research ☐ Other</td><td>Deliberation classification</td><td>New Review</td></tr>
<tr><td>Review Type</td><td colspan="4">Expedited Review</td></tr>
<tr><td>Review Decision</td><td colspan="4">☑ Approval ☐ Expedited Review ☐ Rejection ☐Referral to Full Board Review
Approval after correction and expedited review after correction proceed to expedited review after correction
Supplemental is reviewed at the next regular review after corrections are made</td></tr>
<tr><td>Research Period</td><td colspan="4">April 16, 2024 – February 28, 2026</td></tr>
<tr><td>Approval validity Period</td><td colspan="4">When extending the research period, a change of research plan application is required before the expiration of the approval validity period
April 16, 2024 – April 15, 2025</td></tr>
<tr><td>Submitted documents (ver.4)</td><td colspan="4">• Research plan (human subjects research, human-derived materials research)
• Research consent explanation (human subjects research, human-derived materials research)
Consent exemption statement
Consent document exemption statement
• Response to deliberation opinion
• Bioethics compliance pledge
• Conflict of interest disclosure statement
• Change comparison table
• Human-derived materials(test subject) management ledger
• Research tools such as questionnaires, interviews, and interview questionnaires
• Research subject recruitment documents
• Transfer of materials related to human-derived materials research</td></tr>
<tr><td>Review Comments</td><td colspan="4">[Recommendations]-None
[Revision Requests]- None
[Final judgment]-Approvals
[Justification for Decision]-This study judged “Approved” because the requested modification are appropriate
[Instructions}
- Please note that the consent form for human subject research can be printed from the website of the Bioethics Committee.
- Please apply for a continuation review(interim report) with the committee at least one month before the expiration of the research authorization period
* Before conducting human-derived material research (blood collection) on 100 patients registered at Yeungnam University Hospital, you must obtain approval from the Yeungnam University Hospital Bioethics Committee and submit the approval form through ongoing review(interim report)</td></tr>
<tr><td>Review Date</td><td colspan="2">April 12, 2024</td><td>Continuing Review Period</td><td>Submission deadline: 1 month before the expiration of the approval period
12 months</td></tr>
</table>

This notification informs you that the Institutional Review Board has reviewed your research project and made the above decision.

April 16, 2024
Chair of Yeungnam Univerisity Institutional Review Board

All researchers approved by the institutional committee must comply with the following:

1. If the review result is approval, you may conduct research according to the research plan. However, you may only use the approved consent form.

2. If the review result is expedited review after revision, you may research if you submit a response to the review opinion and the revised content using the e-IRB system and if it is approved through expedited review. However, if the project is not submitted within 6 months, the application will be cancelled.

3. If the review result is supplementation (regular re-review), you may research if you submit a response to the review opinion and the revised content using the e-IRB system and if it is approved through regular review. However, if the project is not submitted within 6 months, the application will be cancelled. 4. If the review result is rejected, you can re-examine the research plan and apply for a new review using the e-IRB system.

5. The research director should receive a review of the research plan and the research completion and research results report review before the review period (expiration date of the research approval period) and upon completion of the research. Review applications should be made at least 1 month before the review period (expiration date of the research approval period), and a research completion report should be submitted to the committee within 3 months after the completion of the research.

6. The research director should report to the committee any serious adverse reactions, violations of the research plan, or unexpected problems.

7. If you have any objections to the review results, please write an objection application form [Appendix 22] and submit it by email (yuirb@yu.ac.kr) within 15 days from the date of notification of the review results. However, re-reviews will not be conducted more than twice for the same matter.

8. Records related to research must be kept for at least three years from the date of completion of the research, following relevant laws.

9. Important Reminders for All Approved Researchers

1) Conduct research according to the approved protocol.
2) Use only the approved consent forms.
3) Obtain prior IRB approval for any protocol changes unless necessary for participant safety.
4) Immediately report emergency modifications.
5) Report any deaths, hospitalizations, or serious illnesses of participants.
6) Report any new information that may affect participant safety.
7) Submit reports as requested by the IRB.
8) Cooperate with IRB inspections.
9) Obtain IRB approval for recruitment advertisements prior to use.
10) Ensure informed consent is obtained voluntarily and without coercion.
11) Implement all required modifications before conducting the research.
12) Submit responses to modification requests within six months.
13) Apply for continuing review at least one month before the approval period ends.
14) Submit final reports within three months of study completion.
15) Store research records for at least three years after study completion.

<Enforcement Decree of the Bioethics and Safety Act, Article 15, Paragraph 1>
1. Research plan and the results of deliberation by the institutional committee that reviewed the relevant research pursuant to Article 10, Paragraph 3, Subparagraph 1 of the Act (including the revised research plan and deliberation results if changed)
2. Written consent from the research subject pursuant to Article 16, Paragraphs 1 and 2 of the Act or written consent exemption approval from the institutional committee pursuant to Paragraph 3 of the same Article
3. Status of collection, use, and provision of personal information
4. Research completion report including research results, etc. and the results of investigation and supervision by the institutional committee on the progress and results of the research pursuant to Article 10, Paragraph 3, Subparagraph 2 of the Act

The Yeungnam University Bioethics Committee complies with domestic laws related to research, including Bioethics and Safety, and respects international and domestic standards related to bioethics and research ethics.

Yeungnam University Institutional Review Board
IRB Review Notification

<table>
<tr><td>Project Number</td><td>2023-R-1203-004</td><td>IRB approval Number</td><td colspan="2">7002016-A-2025-043</td></tr>
<tr><td>Research Title</td><td colspan="4">(국문) 유전체 검사결과에 따른 운동생활에 의한 건강 증진 예측모델 수립에 있어 운동생활 습관의 효과와 이를 기반한 건강 및 질환예방 관리 서비스 개발
(영문) Development of health and disease prevention managing services based on the effectiveness of exercise lifestyle in establishing a prediction model for health promotion by exercise based on genomic test results.</td></tr>
<tr><td rowspan="3">Principal I</td><td>Name</td><td>Hyunseok Jee</td><td>Affiliation</td><td>Yeungnam/Department of Pysical Education</td></tr>
<tr><td>Position</td><td>Professor</td><td>Contact</td><td>010-4900-3213</td></tr>
<tr><td>Email</td><td colspan="3">Jeehs@ynu.ac.kr</td></tr>
<tr><td>Type of Research</td><td colspan="2">☑ Human Subject Research
☑ Human-derived Materials Research ☐ Other</td><td>Classification</td><td>Change review</td></tr>
<tr><td>Review Type</td><td colspan="4">Expedited Review</td></tr>
<tr><td>Review Decision</td><td colspan="4">☑ Approval ☐ Expedited Review ☐ Rejection ☐Referral to Full Board Review
Approval after correction and expedited review after correction proceed to expedited review after correction
Supplemental is reviewed at the next regular review after corrections are made</td></tr>
<tr><td>Research Period</td><td colspan="4">April 16, 2024 – February 28, 2026</td></tr>
<tr><td>Approval validity Period</td><td colspan="4">When extending the research period, a change of research plan application is required before the expiration of the approval validity period
February 28, 2025 – April 15, 2025</td></tr>
<tr><td>Submitted documents (ver.5)</td><td colspan="4">• Research plan (human subjects research, human-derived materials research)
• Modified documents other than required documents
• Change comparison table and research plan
• Research consent explanation (human subjects research, human-derived materials research)
Consent exemption statement
Consent document exemption statement
• Response to deliberation opinion
• Bioethics compliance pledge
• Conflict of interest disclosure statement
• Change comparison table
• Human-derived materials (test subjects) management ledger
• Research tools such as questionnaires, interviews, and interview questionnaires
• Research subject recruitment documents
• Transfer of materials related to human-derived materials research</td></tr>
<tr><td>Review Comments</td><td colspan="4">[Reason for Decision]
- The amendments submitted for this study do not present any issues related to research ethics; therefore, the study is "Approved."
※ For studies involving patients registered at Yeungnam University Hospital, approval from the hospital's IRB must also be obtained prior to proceeding.</td></tr>
<tr><td>Review Date</td><td>February 27, 2025</td><td>Continuing Review Period</td><td colspan="2">12 months</td></tr>
<tr><td colspan="5">This notification informs you that the Institutional Review Board has reviewed your research project and made the above decision.

Date: February 28, 2025</td></tr>
</table>

All researchers approved by the institutional committee must comply with the following:

1. If the review result is approval, you may conduct research according to the research plan. However, you may only use the approved consent form.

2. If the review result is expedited review after revision, you may research if you submit a response to the review opinion and the revised content using the e-IRB system and if it is approved through expedited review. However, if the project is not submitted within 6 months, the application will be cancelled.

3. If the review result is supplementation (regular re-review), you may research if you submit a response to the review opinion and the revised content using the e-IRB system and if it is approved through regular review. However, if the project is not submitted within 6 months, the application will be cancelled. 4. If the review result is rejected, you can re-examine the research plan and apply for a new review using the e-IRB system.

5. The research director should receive a review of the research plan and the research completion and research results report review before the review period (expiration date of the research approval period) and upon completion of the research. Review applications should be made at least 1 month before the review period (expiration date of the research approval period), and a research completion report should be submitted to the committee within 3 months after the completion of the research.

6. The research director should report to the committee any serious adverse reactions, violations of the research plan, or unexpected problems.

7. If you have any objections to the review results, please write an objection application form [Appendix 22] and submit it by email (yuirb@yu.ac.kr) within 15 days from the date of notification of the review results. However, re-reviews will not be conducted more than twice for the same matter.

8. Records related to research must be kept for at least three years from the date of completion of the research, following relevant laws.

9. Important Reminders for All Approved Researchers

1) Conduct research according to the approved protocol.
2) Use only the approved consent forms.
3) Obtain prior IRB approval for any protocol changes unless necessary for participant safety.
4) Immediately report emergency modifications.
5) Report any deaths, hospitalizations, or serious illnesses of participants.
6) Report any new information that may affect participant safety.
7) Submit reports as requested by the IRB.
8) Cooperate with IRB inspections.
9) Obtain IRB approval for recruitment advertisements prior to use.
10) Ensure informed consent is obtained voluntarily and without coercion.
11) Implement all required modifications before conducting the research.
12) Submit responses to modification requests within six months.
13) Apply for continuing review at least one month before the approval period ends.
14) Submit final reports within three months of study completion.
15) Store research records for at least three years after study completion.

<Enforcement Decree of the Bioethics and Safety Act, Article 15, Paragraph 1>
1. Research plan and the results of deliberation by the institutional committee that reviewed the relevant research pursuant to Article 10, Paragraph 3, Subparagraph 1 of the Act (including the revised research plan and deliberation results if changed)
2. Written consent from the research subject pursuant to Article 16, Paragraphs 1 and 2 of the Act or written consent exemption approval from the institutional committee pursuant to Paragraph 3 of the same Article
3. Status of collection, use, and provision of personal information
4. Research completion report including research results, etc. and the results of investigation and supervision by the institutional committee on the progress and results of the research pursuant to Article 10, Paragraph 3, Subparagraph 2 of the Act

The Yeungnam University Bioethics Committee complies with domestic laws related to research, including the Bioethics and Safety , and respects international and domestic standards related to bioethics and research ethics.

Yeungnam University Institutional Review Board
IRB Review Notification

<table>
<tr><td>Project Number</td><td colspan="2">2023-R-1203-005</td><td colspan="2">IRB approval Number</td><td>7002016-A-2025-066</td></tr>
<tr><td>Research Title</td><td colspan="5">(국문) 유전체 검사결과에 따른 운동생활에 의한 건강 증진 예측모델 수립에 있어 운동생활 습관의 효과와 이를 기반한 건강 및 질환예방 관리 서비스 개발
(영문) Development of health and disease prevention managing services based on the effectiveness of exercise lifestyle in establishing a prediction model for health promotion by exercise based on genomic test results.</td></tr>
<tr><td rowspan="3">Principal Investigator</td><td>Name</td><td>Hyunseok Jee</td><td>Affiliation</td><td colspan="2">Yeungnam/Department of Physical Education</td></tr>
<tr><td>Position</td><td>Professor</td><td>Contact</td><td colspan="2">010-4900-3213</td></tr>
<tr><td>Email</td><td colspan="4">Jeehs@ynu.ac.kr</td></tr>
<tr><td>Type of Research</td><td colspan="3">☑ Human Subject Research
☑ Human-Derived Materials Research ☐ Other</td><td>Classification</td><td>Continuing Review</td></tr>
<tr><td>Review Type</td><td colspan="5">Expedited Review</td></tr>
<tr><td>Review Decision</td><td colspan="5">☑ Approval ☐ Expedited Review ☐ Rejection ☐Referral to Full Board Review
Approval after correction and expedited review after correction proceed to expedited review after correction
Supplemental is reviewed at the next regular review after corrections are made</td></tr>
<tr><td>Research Period</td><td colspan="5">April 16, 2024 – February 28, 2026</td></tr>
<tr><td>Approval validity Period</td><td colspan="5">When extending the research period, a change of research plan application is required before the expiration of the approval validity period
April 11, 2025 – April 14, 2026</td></tr>
<tr><td>Submitted documents (ver.1)</td><td colspan="5">• Research plan
• Research consent explanation (human subjects research, human-derived materials research)
Consent exemption statement
Consent document exemption statement
• Copies of signed consent forms from participants
• Other materials provided to participants (e.g., questionnaires)
• Transfer of materials related to human-derived materials research</td></tr>
<tr><td>Review Comments</td><td colspan="5">[Revision Requests]- None
[Recommendations]-None
[Justification for Decision]- Determined that the research was conducted in accordance with the previously approved protocol. Thus, Approved</td></tr>
<tr><td>Review Date</td><td colspan="2">April 10, 2025</td><td colspan="2">Continuing Review Period</td><td>Submission deadline: 1 month before the expiration of the approval period
12 months</td></tr>
</table>

This notification informs you that the Institutional Review Board has reviewed your research project and made the above decision.

April 11, 2025

Chair of Yeungnam Univerisity Institutional Review Board

All researchers approved by the institutional committee must comply with the following:

1. If the review result is approval, you may conduct research according to the research plan. However, you may only use the approved consent form.

2. If the review result is expedited review after revision, you may research if you submit a response to the review opinion and the revised content using the e-IRB system and if it is approved through expedited review. However, if the project is not submitted within 6 months, the application will be cancelled.

3. If the review result is supplementation (regular re-review), you may research if you submit a response to the review opinion and the revised content using the e-IRB system and if it is approved through regular review. However, if the project is not submitted within 6 months, the application will be cancelled. 4. If the review result is rejected, you can re-examine the research plan and apply for a new review using the e-IRB system.

5. The research director should receive a review of the research plan and the research completion and research results report review before the review period (expiration date of the research approval period) and upon completion of the research. Review applications should be made at least 1 month before the review period (expiration date of the research approval period), and a research completion report should be submitted to the committee within 3 months after the completion of the research.

6. The research director should report to the committee any serious adverse reactions, violations of the research plan, or unexpected problems.

7. If you have any objections to the review results, please write an objection application form [Appendix 22] and submit it by email (yuirb@yu.ac.kr) within 15 days from the date of notification of the review results. However, re-reviews will not be conducted more than twice for the same matter.

8. Records related to research must be kept for at least three years from the date of completion of the research, following relevant laws.

9. Important Reminders for All Approved Researchers

1) Conduct research according to the approved protocol.
2) Use only the approved consent forms.
3) Obtain prior IRB approval for any protocol changes unless necessary for participant safety.
4) Immediately report emergency modifications.
5) Report any deaths, hospitalizations, or serious illnesses of participants.
6) Report any new information that may affect participant safety.
7) Submit reports as requested by the IRB.
8) Cooperate with IRB inspections.
9) Obtain IRB approval for recruitment advertisements prior to use.
10) Ensure informed consent is obtained voluntarily and without coercion.
11) Implement all required modifications before conducting the research.
12) Submit responses to modification requests within six months.
13) Apply for continuing review at least one month before the approval period ends.
14) Submit final reports within three months of study completion.
15) Store research records for at least three years after study completion.

<Enforcement Decree of the Bioethics and Safety Act, Article 15, Paragraph 1>
1. Research plan and the results of deliberation by the institutional committee that reviewed the relevant research pursuant to Article 10, Paragraph 3, Subparagraph 1 of the Act (including the revised research plan and deliberation results if changed)
2. Written consent from the research subject pursuant to Article 16, Paragraphs 1 and 2 of the Act or written consent exemption approval from the institutional committee pursuant to Paragraph 3 of the same Article
3. Status of collection, use, and provision of personal information
4. Research completion report including research results, etc. and the results of investigation and supervision by the institutional committee on the progress and results of the research pursuant to Article 10, Paragraph 3, Subparagraph 2 of the Act

The Yeungnam University Bioethics Committee complies with domestic laws related to research, including Bioethics and Safety, and respects international and domestic standards related to bioethics and research ethics.
