# Supplementary material for: The protocol for developing health and disease prevention services: An exercise-based prediction model integrating genomic test results
Source: PLoS One. 2025 Jul 22;20(7):e0327947. doi: 10.1371/journal.pone.0327947 (PMC12282888; doi:10.1371/journal.pone.0327947)
Supplement: S1 File — S1 SPIRIT checklist. S2 Recruitment of research participants. S3 Yeungnam University Research Participant Recruitment Poster. S4 Leaflet Brochure. S5 3 banners. S6 the study plan translator. S7 IRB Review Notification translator. S8 the funding certification. S9 Human Subjects Research Consent Explanation and Consent Form. S10 Medical history questionnaire. S11 Exercise participation questionnaire. (ZIP) [file pone.0327947.s001.zip › S8 the funding certification.pdf]

# CERTIFICATE

Applicant: Hyunseok Jee

Date of Issue: 03.18.2025

Confirmed by: Beomjin Jang(S&T Academic Research Team)

| | | |
|---|---|---|
| Program Title | Research Staff Program | |
| Research Title | The optimal exercise intervention based skeletal muscle derived cancer suppressor and big data used exercise program for the preventive and bettering cancer symptoms | |
| NRF Grant No. | 2021R1I1A3047088 | |
| Funding Scale | 465,548,000KRW(total) | |
| Funding Duration | 06.01.2021~05.31.2026 | |
| Project Investigator (PI) | Name in Full | Hyunseok Jee |
| | Date of Birth | 07.27.1974 |
| | Affiliated Organization | Yeungnam University |
| Purpose | Submission to an organization | |

This is to certify that the above mentioned facts are true and correct.

**National Research Foundation of Korea**

National Research Foundation of Korea(NRF)
Headquarters: 201 GAJEONG-RO, YUSEONG-GU, DAEJEON 34113 KOREA / TEL.82-42-869-6114
Seoul Office : 25 HEOLLEUNG-RO, SEOCHO-GU, SEOUL 06792 KOREA / TEL.82-2-3460-5500
