# Supplementary material for: The protocol for developing health and disease prevention services: An exercise-based prediction model integrating genomic test results
Source: PLoS One. 2025 Jul 22;20(7):e0327947. doi: 10.1371/journal.pone.0327947 (PMC12282888; doi:10.1371/journal.pone.0327947)
Supplement: S1 File — S1 SPIRIT checklist. S2 Recruitment of research participants. S3 Yeungnam University Research Participant Recruitment Poster. S4 Leaflet Brochure. S5 3 banners. S6 the study plan translator. S7 IRB Review Notification translator. S8 the funding certification. S9 Human Subjects Research Consent Explanation and Consent Form. S10 Medical history questionnaire. S11 Exercise participation questionnaire. (ZIP) [file pone.0327947.s001.zip › S9 Human subjects Research Consent Explanation and Consent Form.pdf]

Human Subjects Consent Statement

Version : 1.3

Study Title	(Korean) 유전체 검사결과에 따른 운동생활에 의한 건강 증진 예측모델 수립에 있어 운동생활습관의 효과와 이를 기반한 건강 및 질환예방 관리 서비스 개발			
	(영문) Development of health and disease prevention managing services based on the effectiveness of exercise lifestyle in establishing a prediction model for health promotion by exercise based on genomic test results.			
Principal Investigator	Name	Hyunseok Jee	Affiliations and Status	Physical Education Department/ Associate Professor
Researchers	Name	Yunsuk Choi	Email	genchem@naver.com
	Address	Cheonma Gymnasium 103 room	Phone Number	053-810-3148

This is a study to learn about your health risk through genomic information-based health risk analysis, and to see if this information can help you improve your health by engaging in appropriate exercise in your daily life. Before you decide **whether or no to participate** in this study, you should carefully read the information sheet and consent form, which explain why this study is being conducted, what it will do, and that you can stop participating in this study at any time.

The researcher conducting this study will explain this study to you. This study will only be conducted if you volunteer to participate. Please read the following carefully before deciding if you want to participate, and discuss it with family and friends if necessay.

If you have an questions about participating in the study, your researcher will be able to explain in detail.

Your signature means that you have been informed about the study and its risks, and your signature on this document means that you (or your legal representative) wish to participate in the study. If you have any questions about the ethical aspects of the research or the protection of human subjects's rights, you may contact the University's Institutional Review Board (contact number : 053-810-1177, email:yuirb@yu.ac.kr)

1. The purpose of human subjects research

Knowing your genes-the encoded blueprints that determine your biological features and traits-is tied to a healthy life. A healthy life can be defined as a life free of disease, but real health is achieved when we become more proactive rather than passive in our pursuit of health. The occurrence of disease can be predicted in advance by analyzing the genome, but genetic information alone is not enough to maintain and promote true health because it depends on a multilayered interaction of environmental factors and lifestyle habits in addition to genes that are determined at birth.

So, you could say that genomic analytics can help you understand your health risks and ideally, make predictions to avoid fure misfortune.

Exercise can have mental as well as physical health benefis. However, there are many opinions about the right way to exercise,so it's important to get the most out of your workout based on your genomic results.
The best way to prevent and manage my disease is to anticipate my body's condition in advance and improve my health in the most natural and accurate way through exercise, rather than through surgery or medication with side effects.
Therefore, the purpose of this study is to understand the impact of knowing your health risk based on your genomic information and incorporating appropriate exercise imterventions into your daily routine to improve your health. The goal of this study is to understand how changes in genetic markers for various chronic disease, including cancer, can be altered through evidence-based appropriate exercise.

2. The duration, prodedures, and methods of human subject participation in the study

If you are willing to participate the study will be conducted as follows: 1. Blood(5m), 2.Urine(100ml), or 3. A small amout of oral mucosa using a cotton swab (1 to 3 choices) will be collected three times in toatl at the Department of Exercise Physiology at Yeungnam University, at the sports center you are attending, or at Yeungnam University Hospital in the case of blood, and at the respective sports center in the case of exercise interventions. If the results of the oral mucosa analysis are invalid, it can be re-collected.
You will also be asked to fill out a questionnaire regarding medical history questions. when performing the exercises using the app, you will perform them as described in the app.
You will be contacted individually for this study and will take part in the experiment at a time that is convenient for you. Your participation is expected to last three months.

3. Anticipated risks and benefits to research subjects

No side effects have been reported so far in this study, but if you experience fatigue during the study, you can stop and there will be o penalty for doing so.
As part of your participation in this study, you will receive a detailed explanation of the results of your genomic analysis and related materials. This is a genetic screening of your body that will help prevent disease in individuals by providing opportunity to detect genetic risk factors and respond to then early.
In terms of monetary rewards, you';; likely see analytics cost savings of nearly 50%.

4. Privacy

This study will use the following personal information about you (name, contact details). This information will be used for the duration of the study and the information collected will be managed appropriately in accordance with the Privacy Act. In addition, information about the research subjects obtained form the study will be kept on a locked personal cmputer and will only be accessible to the principal investigator. Every effort will be made to ensure the confidentiality of all personal information obtained through the study.

5. Compensation for losses resulting from participation in the study

If you are injured as a result of your participation in the study, the cost of treatment for your injury will be paid by the Principal investigator.

6. Providing Personal Information

When the data obtained form this study is published in the form of reports or journals, personal information that could identify you will be deleted or anonymized. However, your personal information may be provided if required by law. By signing this consent form, you will be deemed to have been informed of this and to be willing to allow it. The content collected (questionnaires, research consent forms) will be stored appropriately and in accordance with privacy laws(locked boxes or filing cabinets), and the contents of the research collectin will be stored for three years after the end of the study and then destroyed by shredding or incineration. In addition, monitoring personnel, inspection personnel, and the Institutional Review Board will be allowed to inspect the research results in order to verify the relability of the procedures and materials used in this study without violating the confidentiality of the research subjects and within the scope of relevant regulations.

7. Withdrawing consent

Your decision to participate in this study is voluntary, and you may decline to participate at your own discretion. You may also withdraw from the study at any time after you have participated in the study, and you will not be penalized or discriminated against for doing so. In addition, immediately after you inform us of your decision to withdraw, the recorded data will be irretrievably destryed to protect your privacy. if you have any questions about the study that you do not understand, please do not hesitate to ask. Please review the purpose of this study and the consent form thoroughly before deciding whether or not to participate in the study.

Human Subjects Consent Form

Study title	(Korean) 유전체 검사결과에 따른 운동생활에 의한 건강 증진 예측모델 수립에 있어 운동생활습관의 효과와 이를 기반한 건강 및 질환예방 관리 서비스 개발			
	(English)Development of health and disease prevention managing services based on the effectiveness of exercise lifestyle in establishing a prediction model for health promotion by exercise based on genomic test results.			
Principal Investigation	Name	Hyunseok Jee	Affiliations and status	Physical Education Department/ Associate Professor
Researchers	Name	Yunsuk Choi	Email	genchem@naver.com
	Address	Cheonma Gymnasium 103 room	Phone Number	053-810-3148

1. I have a good understanding of the purpose of the study.

2. I was given ample opportunity for clarification and questions.

3. I understand the expected risks and benefits.

4. I am aware of the content of the personal data provided to the research, how it is managed, that it is available to researcher and others, and when it will be disposed of.

5. I understand that I may withdraw my consent to participate in the study and provide personal information at any time (even after the study has ended) and that I will not be penalized for doing so.

6. I voluntarily agree to participate in the above study.

Year Month Day

Principal Investigator : ________________ (Signature)

Year Month Day

Research Subject : ________________ (Signature)

Year Month Day

Legal Representative : ________________ (Signature
