# Supplementary material for: The protocol for developing health and disease prevention services: An exercise-based prediction model integrating genomic test results
Source: PLoS One. 2025 Jul 22;20(7):e0327947. doi: 10.1371/journal.pone.0327947 (PMC12282888; doi:10.1371/journal.pone.0327947)
Supplement: Attachment — Submitted filename: renamed_45382.docx [file pone.0327947.s002.docx]

Subject: Response to Editorial Requests – Manuscript ID [PONE-D-25-10857]

Thank you for your thoughtful review and guidance on our manuscript. We appreciate the opportunity to revise and improve our submission. Below, we provide our responses to the requested revisions:

1. SPIRIT Schedule as Figure 1:

- We have re-uploaded the SPIRIT schedule as Figure 1, ensuring that it aligns with the journal’s submission guidelines.
- The figure legend and captions have been updated accordingly in both the manuscript and Supporting Information.

2. Title Revision: The Developing health and Disease Prevention Services: An Exercise-Based Prediction Model Integrating Genomic Test Results

- As requested, we have updated the manuscript title to include the word "protocol."
- The title has also been revised in PLOS ONE’s manuscript data/title section to maintain consistency.

3. SPIRIT Schedule Placement:

- We have confirmed that the SPIRIT schedule is now assigned as Figure 1 and has been correctly labeled throughout the manuscript.
- We have also renamed the corresponding Supporting Information file and updated the references to Figure 1 in manuscript page 20 and the Supporting Information section.

4. English Translation of the Study Protocol:

- The English-translated version of the approved study protocol has been included as a Supporting Information file.
- This file is located in the compressed Supporting Information folder (S6: The Study Plan Translator).

We appreciate your time and consideration and look forward to your feedback. Please let us know if any additional modifications are required.
